# Adjustment for tobacco smoking and alcohol consumption by simultaneous analysis of several types of cancer

**DOI:** 10.1007/s10552-016-0847-x

**Published:** 2017-02-02

**Authors:** Tor Haldorsen, Jan Ivar Martinsen, Kristina Kjærheim, Tom K. Grimsrud

**Affiliations:** Department of Research, Cancer Registry of Norway, Pb 5313, Majorstuen, 0304 Oslo, Norway

**Keywords:** Alcohol drinking, Bias, Confounding factors, Epidemiological methods, Neoplasms, Tobacco smoking

## Abstract

**Purpose:**

Tobacco smoking and alcohol consumption are risk factors for several types of cancer and may act as confounders in aetiological studies. Large register-based cohorts often lack data on tobacco and alcohol. We present a method for computing estimates of cancer risk adjusted for tobacco and alcohol without exposure information.

**Methods:**

We propose the use of confirmatory factor analysis models for simultaneous analysis of several cancer sites related to tobacco and alcohol. In the analyses, the unobserved pattern of smoking habits and alcohol drinking is considered latent common factors. The models allow for different effects on each cancer site, and also for appropriate latent site-specific factors for subgroup variation. Results may be used to compute expected numbers of cancer from reference rates, adjusted for tobacco smoking and alcohol consumption. This method was applied to results from a large, published study of work-related cancer based on census data (1970) and 21 years of cancer incidence data from the national cancer registry.

**Results:**

The results from our analysis were in accordance with recognised risks in selected occupational groups. The estimated relative effects from tobacco and alcohol on cancer risk were largely in line with results from Nordic reports. For lung cancer, adjustment for tobacco implied relative changes in SIR between a decrease from 1.16 to 0.72 (Fishermen), and an increase from 0.47 to 0.95 (Forestry workers).

**Conclusions:**

We consider the method useful for achieving less confounded estimates of cancer risk in large cohort studies with no available information on smoking and alcohol consumption.

**Electronic supplementary material:**

The online version of this article (doi:10.1007/s10552-016-0847-x) contains supplementary material, which is available to authorized users.

## Introduction

Tobacco smoking and alcohol consumption are related to several types of cancer [1] and constitute major risk factors, alone or in combination, for many of them. Unevenly distributed consumption of tobacco and alcohol may, therefore, seriously hamper the identification of other causal factors in the absence of appropriate confounder control, which may be the case in large studies based on linkage between census data and cancer registries [2, 3]. For decades, methods for control of tobacco smoking in occupational studies have been discussed [4–6] and evaluated [7–10]. Others have assessed the effect of controlling for tobacco and alcohol at the same time [11, 12]. The need for confounder control and bias assessment may vary according to scientific challenge or regulatory questions, but observed variation in cancer risk between regions, over time, and between populations does, indeed, demonstrate that the issue is of some concern [13–17].

In general, the effects from tobacco and alcohol are substantial and exceed those from most occupational exposures [18, 19]. This dominance makes it probable that within any occupational group, roughly, the same relative incidence will be seen between the tobacco related cancer sites. This is also to be expected for the group of alcohol-related cancers. Some deviation from this pattern may be caused by occupational exposures. Since tobacco and alcohol relate to each cancer site to a different degree and a number of cancer sites are related to both agents, we propose a quantitative method for obtaining confounder adjustment based on the observed cancer incidence pattern. The method is based on models for confirmatory factor analysis [20, 21] and as an illustration, it is applied to published data on Norwegian men in a study of occupation and cancer [2].

Standardised incidence ratios (SIRs) are a measure of the relative rate of cancer in a study group compared with a reference, adjusted (‘standardised’) for age distribution in the study group. SIRs are obtained by dividing the numbers of observed incident cancer cases in the study group by the ‘expected numbers’, which have been derived by multiplying person-time in the study group cross-classified by 5-year age strata and calendar periods with corresponding age- and period-specific incidence rates from the reference population. A serious limitation in the interpretation of SIRs is the lack of adjustment for potential strong confounders, and the reference rates are calculated from a mixture of people with different smoking and drinking habits. Our aim was to present a method for computing SIRs adjusted for tobacco smoking and alcohol consumption without access to explicit information on these exposures.

## Methods

We chose to apply the method on results from a published study on Norwegian men, derived from a Nordic investigation of work-related cancer [2]. The Norwegian cohort was established by information from the national census of 1 November 1970, and the men were followed for cancer incidence, according to 54 occupational groups, from 1971 to 1991 by linkage to the national cancer registry. The Norwegian part involved 893,264 men and 16,851,687 person-years. Details on the material and results are found elsewhere [2].

For this study, we used the incidence rates of eight cancer sites related to tobacco and alcohol in combination (tongue, mouth, pharynx, oesophagus, larynx, liver, colon, and rectum) [1]. Another eight sites were related to tobacco only (lung, bladder, kidney, pancreas, nose, stomach, acute leukaemia, and other types of leukaemia) [1], but we disregarded acute leukaemia and other types of leukaemia, as preliminary analyses indicated scarcely any variation in risk by occupation for these cancers. We, therefore, addressed 14 cancer sites in 52 occupational groups (inclusive of a group of economically inactive). Two occupational groups addressed in the former study were not included here due to very small numbers. Basic statistics for the present data is presented in Table 1.

**Table 1:** Summary statistics on (a) number of cancer cases, and (b) person-years by age on 1 January 1971 in a study of men according to 52 occupational groups from the Norwegian national 1970 census, followed for cancer 1971–1991

(a)				(b)	
Cancer site	Mean	Minimum	Maximum	Age (years)	Person-years
Stomach	146.9	1	801	25–64	16,844,123
Pancreas	75.5	3	350	25–29	2,702,740
Nose	6.5	0	39	30–34	2,113,562
Lung	305.2	8	1,549	35–39	1,979,385
Kidney	68.2	1	289	40–44	2,176,681
Bladder	163.5	4	700	45–49	2,372,849
Tongue	9.7	0	92	50–54	2,210,925
Mouth	13.5	0	78	55–59	1,862,012
Pharynx	16.2	0	126	60–64	1,425,969
Oesophagus	25.3	0	170		
Colon	172.0	2	780		
Rectum	119.5	1	565		
Liver	15.7	0	119		
Larynx	30.4	1	153		

The two groups of cancers were analysed separately. For each of them, we applied confirmatory factor analysis models with latent common factors for the unobserved exposures to tobacco only and to tobacco and alcohol combined, respectively, and appropriate latent site-specific factors for the occupational variation on each cancer type [22]. An alternative would be to analyse all 14 sites simultaneously with two latent common factors, one for tobacco and one for alcohol, but, since no site was related only to alcohol this solution could lead to numerical instability.

Basic components of our models were labelled by occupational group, *i* = 1, 2,…, 52; and cancer site, *j* = 1, 2,…, 6 or 8; so that observed and ‘expected cases’ from the data set were denoted with *X*_*ij*_ and *E*_*ij*_, respectively. Let *L*_*i*_ and *U*_*ij*_, respectively, be values for the occupational group *i* on the common factor, and on the site-specific factor for cancer site *j*. For each cancer site *j*, it is assumed a latent structure of risk composed of a linear function of the common factor (alcohol and/or tobacco), and possibly a site factor (occupational variation). In the linear function, the constant is denoted *a*_*j*_ and the slope (factor loading) is denoted *b*_*j*_. It is assumed that *L*_*i*_ and *U*_*ij*_ are normally distributed with means = 0.0 and that each site factor is independent of the common factor. With given values of common and site factors and parameter values for *a*_*j*_ and *b*_*j*_, it is assumed that the conditional distribution of *X*_*ij*_ is Poisson with expectation ${{H}_{ij}}~={{E}_{ij}}\text{ }\!\!~\!\!\text{ }\times \text{ }\!\!~\!\!\text{ exp}({{a}_{j}}\text{ }\!\!~\!\!\text{ }+\text{ }\!\!~\!\!\text{ }({{b}_{j}}\times {{L}_{i}})+{{U}_{ij}})$.

For the cancers related to tobacco but not to alcohol, we interpreted the common factor as a tobacco score representing the deviation from the population mean for each occupational group, and for the cancers related to both tobacco and alcohol, we interpreted the common factor as a score for the combined effect of tobacco smoking and alcohol consumption. The common factors may be predicted (estimated) for each occupation and give a relative measure for the burden of exposure. The product of the scores of the common factors and the estimated factor loading indicate the relative effect on each type of cancer. The scores of the latent site-specific factors were indicating an occupational variation in risk for the relevant type of cancer.

We started the search for an adjustment model with only the common factor included, and added statistically significant site factors in a stepwise manner. Statistically significant covariances between site factors were also included. Components of the model were re-evaluated on each step.

In the final models, estimates of factor loadings were evaluated by informal comparison to reported relative cancer risks in users of alcohol and/or tobacco *versus* never users for each type of cancer. The inclusion of site-specific factors was checked with what is known from the literature on differences in cancer risk between occupations. The relative fit of final models was compared to baseline models by Akaike’s Information Criterion (AIC) [23]. For our sample size (*n* = 52), it has been proposed that a decrease in AIC of more than 9.0 indicates an improved model [23, p. 119].

From the final models, predicted values for the common factors were computed by empirical Bayes’ means [20, 24] and used together with the estimated factor loadings to compute adjusted expected values according to the formula ${{\hat{F}}_{ij}}\text{= }{{E}_{ij}}\times \text{exp}(\text{ }\!\!~\!\!\text{ }{{\hat{a}}_{j\text{ }\!\!~\!\!\text{ }}}+({{\hat{b}}_{j}}\text{ }\!\!~\!\!\text{ }\times {{\hat{L}}_{i\text{ }\!\!~\!\!\text{ }}}))$ for *i* = 1, 2,…, 52 (occupation) and *j* = 1, 2,…, 6 or 8 (cancer site). Adjusted SIRs were computed by the formula $\text{adjSIR }\!\!~\!\!\text{ }=\text{ }\!\!~\!\!\text{ }{{X}_{ij}}\text{/}{{\hat{F}}_{ij}}.$ The relative bias without adjustment (degree of confounding by alcohol and/or tobacco) was computed by $({\text{SIR}} - {\text{adjSIR}})/{\text{adjSIR}} = (\hat{F}_{{ij}} /E_{{ij}} )-1$.

Programs in Stata 13 were used in the analysis [25]. The program for Generalized Structural Equation Modeling (GSEM) was used for estimation in the basic models [20]. We used two-sided tests for statistical significance and a significance level of 0.05.

## Results

For the factor loadings and all the site factors included in the model, the results for cancer related to tobacco smoking only are presented in Table 2. All cancer sites had statistically significant positive factor loadings on the common factor (‘Tobacco’). Lung cancer was chosen to anchor the factor loadings by setting its value equal to 1.0. All other cancer sites had smaller factor loadings, with the smallest seen for kidney cancer, 0.26 [95% confidence interval (95% CI) 0.12, 0.40]; and bladder cancer, 0.31 (95% CI 0.18, 0.44). Results in-between were found for cancer of the stomach, pancreas, and nose. The estimate for variance of the common factor (‘Tobacco’) was 0.104 (95% CI 0.061, 0.177). The highest estimated variance among the site factors was for lung cancer, 0.019 (95% CI: 0.006, 0.055). Analysis revealed that there was a perfect correlation between the site factors for kidney cancer and bladder cancer. A site factor for bladder/kidney anchored to bladder cancer was included in the model to reflect this. There was a negative covariance between the site factors for stomach and bladder/kidney. AIC was 2313.01 for the model chosen for adjustment and 2406.59 for the baseline model (independence). AIC was 2400.73 for the common-factor-only model.

**Table 2:** Estimates in factor analysis model (unstandardised) for six cancer sites related to tobacco smoking in a study of 52 occupational groups of men from the Norwegian national 1970 census, followed for cancer 1971–1991

Description of estimate	Estimates	95% CI^a^
Effect of latent factor ‘Tobacco’ on cancer incidences (factor loadings)		
(1) Stomach	0.52	0.35, 0.69
Constant	−0.01	−0.07, 0.06
(2) Pancreas	0.41	0.27, 0.55
Constant	0.03	−0.02, 0.08
(3) Nose	0.42	0.07, 0.77
Constant	0.02	−0.09, 0.14
(4) Lung	1.00	Fixed
Constant	0.02	−0.08, 0.12
(5) Kidney	0.26	0.12, 0.40
Constant	0.04	−0.01, 0.08
(6) Bladder	0.31	0.18, 0.44
Constant	0.04	0.00, 0.09
Effect of site factor ‘Bladder/Kidney’ on incidence of kidney cancer	0.88	0.32, 1.43
Variances of ‘Tobacco’ common factor and site factors		
Tobacco	0.104	0.061, 0.177
Stomach	0.010	0.004, 0.026
Lung	0.019	0.006, 0.055
Bladder/kidney	0.006	0.002, 0.016
Covariance (stomach, bladder/kidney)	−0.007	−0.013, −0.002

^a^Confidence interval

The results imply that if an occupational group (i) has a value of the common factor (‘Tobacco’) of 0.4, the adjusted expected value for lung cancer equals *E*_i4_ × exp(0.02 + 1.00 × 0.4) = *E*_i4_ × 1.52 (see formula in “Methods”, cancer site subscript *j* = 4 reflects numbering in Table 2); and for kidney cancer, it equals *E*_i5_ × exp(0.04 + 0.26 × 0.4) = *E*_i5_ × 1.15 (subscript *j* = 5 according to Table 2). E_i4_ and E_i5_ are the original unadjusted expected values for lung cancer and kidney cancer, respectively [2].

Factor loadings and all site factors for cancer related to both tobacco smoking and alcohol consumption are presented in Table 3. All cancer sites had statistically significant positive factor loadings on the common factor (‘TobAlc’). Pharynx cancer was chosen to anchor the factor loadings (value 1.0) and cancer of the tongue had an estimated factor loading 1.10 (95% CI 0.85, 1.34). Colon cancer and rectum cancer had the lowest estimated factor loadings, 0.17 (95% CI 0.08, 0.27) and 0.15 (95% CI 0.07, 0.23), respectively. The other cancer sites had higher estimated loadings although below 1.0. The variance of the common factor ‘TobAlc’ was estimated to 0.215 (95% CI 0.128, 0.361). There was a perfect correlation between the site factors for colon and rectum cancer. To reflect this, a colon/rectum factor anchored to colon cancer was introduced. A site factor for larynx cancer was included in the model, its variance was estimated to 0.018 (95% CI 0.005, 0.061). AIC was 2,517.00 for the adjustment model and 2,726.04 for the baseline model. AIC was 2586.88 for the common-factor-only model.

**Table 3:** Estimates in factor analysis model (unstandardised) for eight cancer sites related to both tobacco smoking and alcohol consumption in a study of 52 occupational groups of men from the Norwegian national 1970 census, followed for cancer 1971–1991

Description of estimate	Estimates	95% CI^a^
Effect of latent factor ‘TobAlc’, i.e., the combined effect of tobacco and alcohol, on cancer incidences (factor loadings)		
(1) Tongue	1.10	0.85, 1.34
Constant	0.03	−0.15, 0.20
(2) Mouth	0.74	0.55, 0.93
Constant	0.05	−0.07, 0.17
(3) Pharynx	1.00	Fixed
Constant	0.04	−0.11, 0.19
(4) Oesophagus	0.79	0.63, 0.95
Constant	0.05	−0.07, 0.17
(5) Colon	0.17	0.08, 0.27
Constant	0.04	−0.01, 0.08
(6) Rectum	0.15	0.07, 0.23
Constant	0.03	−0.01, 0.07
(7) Liver	0.74	0.57, 0.92
Constant	0.05	−0.07, 0.17
(8) Larynx	0.74	0.55, 0.93
Constant	0.04	−0.08, 0.16
Effect of site factor colon/rectum on incidence of rectum cancer	0.76	0.43, 1.08
Variances of ‘TobAlc’ common factor and site factors		
TobAlc	0.215	0.128, 0.361
Colon/rectum	0.010	0.005, 0.020
Larynx	0.018	0.005, 0.061

^a^Confidence interval

As for the example from tobacco only, these results imply that if an occupational group (i) has a value of the common factor (‘TobAlc’) of 0.4, the adjusted expected value for pharynx cancer equals *E*_i3_ × exp(0.04 + 1.00 × 0.4) = *E*_i3_ × 1.55 (formula from the “Methods”, cancer site subscript *j* = 3 reflecting the numbering in Table 3); and for rectum cancer, it equals *E*_i6_ × exp(0.03 + 0.15 × 0.4) = *E*_i6_ × 1.09 (subscript *j* = 6 according to Table 3). *E*_i3_ and *E*_i6_ are the original unadjusted expected values for pharynx cancer and rectum cancer, respectively [2].

**Fig. 1:**
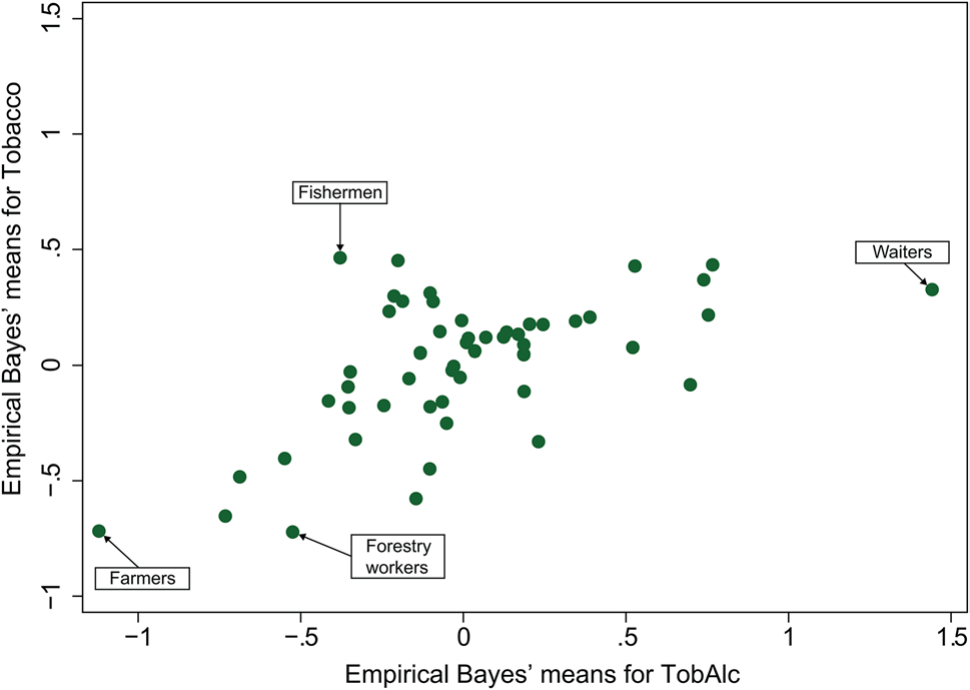
Empirical Bayes’ means of common factors (‘scores’), indicating the effect from tobacco (‘Tobacco’), and tobacco and alcohol (‘TobAlc’), respectively, derived by fitting models for confirmatory factor analysis to incidence data on smoking related cancers, and alcohol- and smoking-related cancers in 52 occupational groups among men in the Norwegian national 1970 census, followed 1971–1991. A score equal to 0.0 is in line with the population mean, while negative or positive scores signify lower or higher scores, respectively

Predicted values (Empirical Bayes’ means) for the two common factors are graphed in Fig. 1, and listed in Supplementary Table 1 with corresponding standard errors. In general, there was a monotonic relationship between the two factors, but there were some occupations with a high tobacco score and a moderate score for the combined effect.

Predicted values for the common factors were used together with results in Tables 2 and 3 to compute adjusted expected values and adjusted SIRs. Fishermen had the highest predicted tobacco score (0.464) and experienced the greater relative reduction when the SIRs were adjusted. For lung cancer, SIR decreased from 1.16 to 0.72, for bladder cancer from 1.17 to 0.97 and for kidney cancer, from 1.18 to 1.01. Forestry workers had the lowest predicted tobacco score (−0.722), and the SIR increased with adjustment from 0.47 to 0.95 for lung cancer, from 0.63 to 0.76 for bladder cancer, and from 0.86 to 1.00 for kidney cancer.

Waiters had the highest predicted value for the latent factor of combined effect of alcohol and tobacco (1.441) and thus experienced the highest relative decrease in SIRs for eight cancer sites when adjusting for this combined factor. SIR decreased from 1.81 to 0.60 for larynx cancer. Farmers had the lowest predicted value (−1.121) and experienced the highest relative increase when adjusting for this combined factor. SIR increased from 0.34 to 0.75 for larynx cancer.

**Fig. 2:**
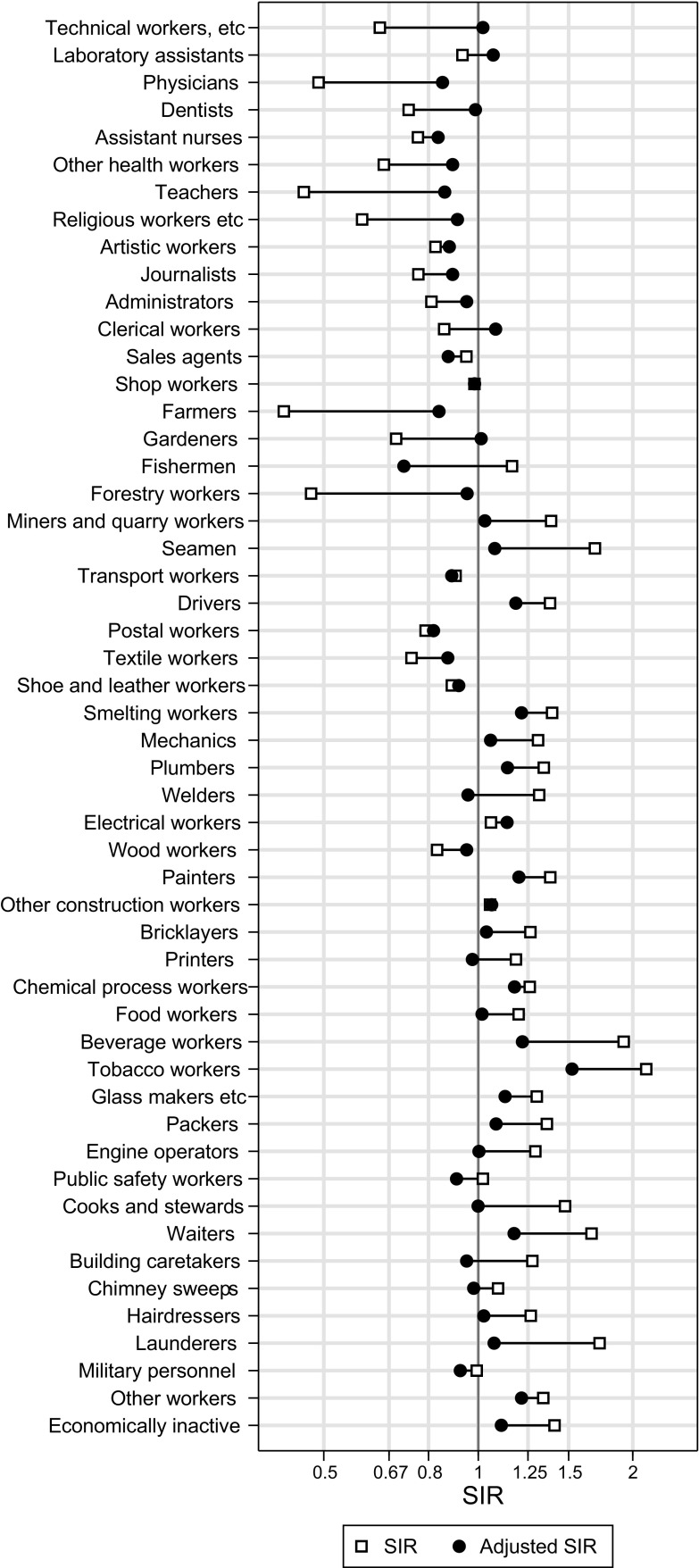
Original standardised incidence ratios (SIR; from Andersen et al. [2]) and tobacco-adjusted SIR for lung cancer plotted for 52 occupational groups among men in the Norwegian national 1970 census, followed 1971–1991

**Fig. 3:**
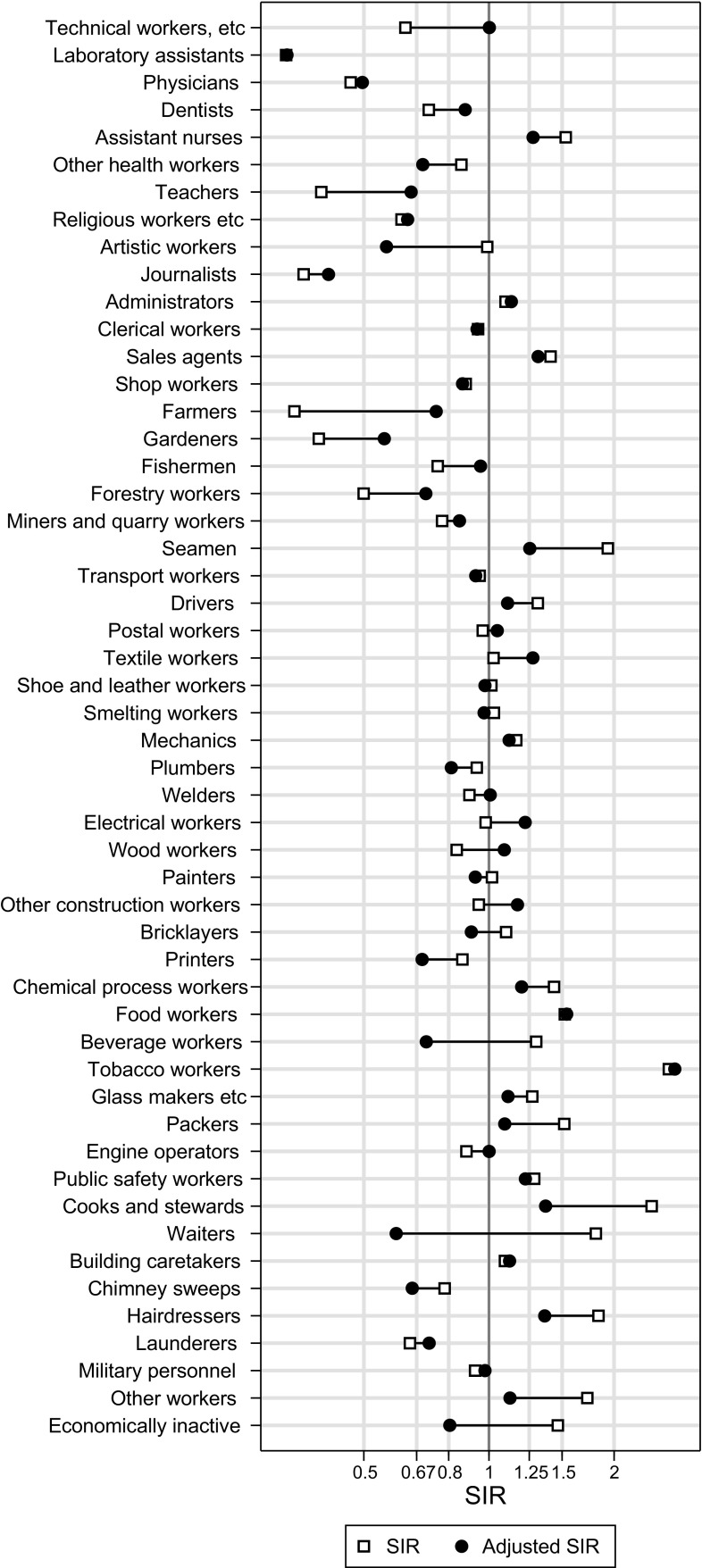
Original standardised incidence ratios (SIR; from Andersen et al. [2]) and tobacco- and alcohol-adjusted SIR for larynx cancer plotted for 52 occupational groups among men in the Norwegian national 1970 census, followed 1971–1991

For lung cancer, original SIRs [2] and adjusted SIRs (adjusted for tobacco) are presented in Fig. 2, and for larynx cancer (alcohol- and tobacco-related), original and adjusted SIRs are presented in Fig. 3. The corresponding SIR values, with 95% confidence intervals for the adjusted ones, are listed in Supplementary Table 2.

For lung cancer, the adjustment decreased the SIR for drivers from 1.38 to 1.18 (95% CI 1.11, 1.26), and for smelting workers from 1.39 to 1.21 (95% CI 1.09, 1.35). For nasal cancer, the SIRs changed with less than 20% for wood workers and for smelting workers (the latter including nickel-refinery workers with a known high nasal cancer risk), both remaining elevated with SIRs of 1.45 and 2.45, respectively.

## Discussion

Our analysis of 14 cancers changed the estimated occupational risks for several sites. The larger changes took place for cancers related to both tobacco and alcohol. The analysis was performed at an aggregated level and was based on models specifying the relationship between risks for several types of cancer. There is a need to compare details of the results with established knowledge to assess the validity of the adjustments.

Our method for adjustment relies on two measurement models. They are examples of generalized structural equation models (GSEM) and their performance will depend on various aspects of fit for the models. We used AIC for relative comparison to baseline models, and for our sample size (*n* = 52), it has been proposed that a decrease in AIC of more than 9.0 indicate a ‘better’ model [23, p. 119]. This was fulfilled comparing the adjustment models to independence models. The criterion was also satisfied if the adjustment models were compared to the simpler common-factor-only models. This does not exclude the existence of ‘better’ models, but from our procedure, we know that the fit cannot be increased (5% level of significance) by adding more site-specific factors.

Evaluation of models is not only a question of statistical measures of fit. Our common factors are indirectly defined by the factor loadings, and we, therefore, checked if the estimates seemed reasonable compared to the known strengths of the relationship between the risk factor and cancer at different sites. To achieve a proper adjustment, the models must also permit other sources of variation than use of alcohol and tobacco, which we did allow for.

The common factors are indirectly defined by the incidence of cancers known to be related to the exposure in question (tobacco alone, or alcohol and tobacco in combination). From studies with individual data, it is recognised that several aspects of the exposure (e.g., exposed or not, duration of exposure, intensity, termination, or different combinations of exposure) may influence individual risk [1]. Furthermore, the effect may vary between cancer types, which opens for complex relationships at the group level. Our adjustment models, however, rely on an assumption that the same measure of exposure may adequately describe the exposure-related effect on the incidence rate of each included cancer type. This assumption is only partly validated in our study, and our results should be taken with caution, because testing of measurement invariance was not conducted in the study.

These reservations made, we did expect a positive relationship between each of the two latent common factors and the incidence of each cancer site. Our expectation was, indeed, fulfilled as all factor loadings were statistically significant and larger than 0.0. A check of the size of the factor loadings is not completely straightforward, since several aspects of smoking and alcohol consumption may influence the individual risk (as pointed out above), but we chose to compare with relative risks reported for smokers *versus* never smokers. The factor loadings for bladder and kidney cancer were 1/3 of that for lung cancer, taken to be reasonably in line with the relative risks from smoking 10–19 cigarettes/day of approximately 12 and 2.5 for lung cancer and urinary tract cancers, respectively [26]. This kept in mind, the estimates for stomach cancer and pancreas cancer were slightly above expected, although the limited precision reflected in the confidence intervals should be considered. This is even more so for the rare cancer of the nose.

Smoking and alcohol consumption are strong risk factors for cancers of the upper aerodigestive tract (tongue, mouth, pharynx, oesophagus, and larynx) when each factor is adjusted for the other one [26, 27]. It is also found that the presence of both factors at the individual level increases the risk more than multiplicatively (synergism) [1]. Correspondingly, high factor loadings for the combined effect were found in our analysis (Table 3), somewhat surprisingly also for liver cancer. The relationship between smoking and liver cancer has been described as modest, and the quantification of the risk related to alcohol as difficult [1]. Smaller loadings for colon and rectum are in line with estimates reported in other studies [28, 29]. The larger variance of the combined factor ‘TobAlc’ (0.215) compared with that of tobacco alone (0.104) (Table 2) was expected, since the former reflects the combined effect of both risk factors.

In the model for cancers related to tobacco only, site-specific factors for stomach cancer, lung cancer, and bladder/kidney cancer were included in the final model. This is in agreement with the notion that lung cancer is the most frequent occupational cancer and bladder cancer possibly is the second most frequent [30–32]. Stomach cancer, on the other hand, is more often linked to socioeconomic factors than to occupational exposures, and its site-specific factor in our model may partly be a result of the close relationship between occupation and socioeconomic status [3].

In the analysis of cancers related both to tobacco and alcohol, we included site-specific factors for colon/rectum and larynx cancer. The first one may be due to differences in other lifestyle factors between occupations, while some workplace exposures have, indeed, been linked to cancer of the larynx [30, 32, 33].

Under the assumption that our adjusted SIRs were correct, we computed the size of the bias in the original SIRs, which for lung cancer varied from 50% too small (negative confounding) to 62% too high (positive confounding). Based on individual smoking data, an occupational mortality study from USA found the unadjusted standardised mortality ratios (SMRs) for lung cancer to be from 35% too low to 43% too high given that the adjusted ones were correct [8]. Based on information on the prevalence of smokers, former smokers, and non-smokers in Finnish occupational groups, the unadjusted SIRs for lung cancer were estimated to be from 33% too low to 31% too high [7]. In the study from USA, unadjusted bladder cancer SMRs were from 16% too low to 13% too high [8], in line with our unadjusted bladder cancer SIRs, suggested to be from 16% too low to 21% too high.

A larger bias was found for most of the cancers related to tobacco and alcohol in combination, a result of the larger variance of the common factor combined with relatively high factor loadings. The high incidence of these cancers among waiters and the low incidence among farmers have been identified earlier in independent studies [3, 34, 35]. Corresponding differences in consumption have been indicated in studies from Norway and neighbouring Sweden [9, 36, 37].

A special problem for the present adjustment is that lung cancer incidence is the best indicator of smoking exposure and, at the same time, the cancer site most heavily linked to occupational exposures. In the search for an adjustment model, it is important to let the data speak for itself and to allow for both sources of variation by checking whether site-specific factors should be included. A site factor was, indeed, included for lung cancer in the adjustment model. We evaluated empirically whether our adjustment led to unduly strong adjustment that would mask the occupational risk in groups known to be exposed to lung carcinogens at work. The adjustment gave only a moderate reduction in the lung cancer SIRs for drivers (from 1.38 to 1.18) and smelting workers (from 1.39 to 1.21), suggesting that overadjustment was not necessarily a consequence of our method.

For nasal cancer, known from studies with individual data to be only weakly related to smoking, the occupational risks among wood workers and smelting workers remained largely unchanged after adjustment, in line with what we would expect.

In an earlier analysis, a completely different method was used to obtain smoking-adjusted lung cancer SIRs on the same set of data [9]. External aggregated information on tobacco smoking habits was assigned to occupational groups and included in a regression model as four parameters. We compared the relative changes in expected values with those in this study, and found a correlation of 0.81, weighted with expected numbers of cases. The former work suggested that the original SIRs for lung cancer were from 50% too low to 50% too high, a result close to that of the present analysis.

Our results suggested that the unadjusted relative risks were somewhat more biased (confounded) than others have found [7, 8, 10, 11, 38]. This may be a result of the scenario for comparison. While we assess the degree of confounding in analyses based on national census data, others have studied confounding in more restricted geographical or socioeconomic settings, possibly with less potential for bias.

We used the incidence rates of groups of cancers to indirectly estimate exposure to alcohol and tobacco. Others have used the incidence of lung cancer alone as a measure of tobacco consumption [39]. Although self-reported consumption data are commonly used, there is evidence to suggest that biochemical markers of tobacco smoking may improve the prediction of lung cancer risk [40–42]. For assessment of confounding on an aggregated level, our biological approach, *via* the observed incidence of tobacco- and/or alcohol-related cancers, could very well be more valid than crude estimates based on imprecise and misclassified reporting of smoking and drinking habits in cross-sectional surveys.

Based on our results, we think that special precaution should be taken when effects of workplace exposures are addressed for cancers related to tobacco and alcohol. One should also remember that inappropriate adjustment for smoking and alcohol consumption may mask a low to moderate occupational risk if the exposures are correlated or act synergistically.

We have used factor analysis models for 14 types of cancer to achieve smoking- and alcohol-adjusted SIRs for occupational groups. Similar methods could also be used in other settings where there is a lack of information on tobacco and alcohol for basic aggregated units of analysis, e.g., regional units of a country.

New statistical methods have emerged in later decades, and the increase in computational power has offered new possibilities for analysis. We have taken advantage of this development in addressing a classical challenge in epidemiology. Formal methods for simultaneous analysis of several cancer sites may be a vehicle for deriving less confounded estimates of cancer risk.

## Electronic supplementary material

Below is the link to the electronic supplementary material.

Supplementary material 1 (DOCX 35 KB)

## Abbreviations

SIR: Standardised incidence ratio
CI: Confidence interval
