# Supplementary material for: Adjustment for tobacco smoking and alcohol consumption by simultaneous analysis of several types of cancer
Source: Cancer Causes Control. 2017 Feb 2;28(2):155–65. doi: 10.1007/s10552-016-0847-x (PMC5306353; doi:10.1007/s10552-016-0847-x)
Supplement: Supplementary file 1 — Supplementary material 1 (DOCX 35 KB) [file 10552_2016_847_MOESM1_ESM.docx]

| **Supplementary Table 1** Empirical Bayes' means of common factors (‘scores’), indicating the effect from tobacco (‘Tobacco’), and tobacco and alcohol (‘TobAlc’), respectively, with standard errors (SE). A score equal to 0.0 is in line with the population mean, while negative or positive scores signify lower or higher scores, respectively | | | | |
| --- | --- | --- | --- | --- |
| Occupational group | Tobacco | SE | TobAlc | SE |
| Technical workers, etc | –0.483 | 0.085 | –0.687 | 0.113 |
| Laboratory assistants | –0.159 | 0.171 | –0.064 | 0.277 |
| Physicians | –0.578 | 0.152 | –0.145 | 0.221 |
| Dentists | –0.321 | 0.182 | –0.332 | 0.302 |
| Assistant nurses | –0.113 | 0.212 | 0.187 | 0.332 |
| Other health workers | –0.330 | 0.172 | 0.232 | 0.247 |
| Teachers | –0.653 | 0.095 | –0.732 | 0.133 |
| Religious workers etc | –0.449 | 0.107 | –0.103 | 0.136 |
| Artistic workers | –0.083 | 0.149 | 0.698 | 0.184 |
| Journalists | –0.174 | 0.178 | –0.244 | 0.299 |
| Administrators | –0.180 | 0.067 | –0.102 | 0.073 |
| Clerical workers | –0.252 | 0.073 | –0.051 | 0.080 |
| Sales agents | 0.061 | 0.072 | 0.035 | 0.079 |
| Shop workers | –0.021 | 0.091 | –0.035 | 0.112 |
| Farmers | –0.718 | 0.056 | –1.121 | 0.080 |
| Gardeners | –0.403 | 0.109 | –0.549 | 0.171 |
| Fishermen | 0.464 | 0.079 | –0.379 | 0.118 |
| Forestry workers | –0.722 | 0.114 | –0.525 | 0.169 |
| Miners and quarry workers | 0.276 | 0.111 | –0.187 | 0.180 |
| Seamen | 0.429 | 0.082 | 0.528 | 0.083 |
| Transport workers | –0.004 | 0.091 | –0.030 | 0.114 |
| Drivers | 0.133 | 0.069 | 0.169 | 0.072 |
| Postal workers | –0.057 | 0.113 | –0.167 | 0.166 |
| Textile workers | –0.183 | 0.115 | –0.352 | 0.177 |
| Shoe and leather workers | –0.052 | 0.155 | –0.009 | 0.253 |
| Smelting workers | 0.116 | 0.095 | 0.016 | 0.125 |
| Mechanics | 0.192 | 0.062 | –0.005 | 0.066 |
| Plumbers | 0.142 | 0.115 | 0.133 | 0.160 |
| Welders | 0.298 | 0.104 | –0.214 | 0.156 |
| Electrical workers | –0.093 | 0.084 | –0.354 | 0.110 |
| Wood workers | –0.154 | 0.058 | –0.415 | 0.069 |
| Painters | 0.119 | 0.100 | 0.069 | 0.135 |
| Other construction workers | –0.029 | 0.074 | –0.348 | 0.098 |
| Bricklayers | 0.176 | 0.110 | 0.204 | 0.152 |
| Printers | 0.175 | 0.117 | 0.245 | 0.160 |
| Chemical process workers | 0.047 | 0.090 | 0.185 | 0.107 |
| Food workers | 0.144 | 0.089 | –0.071 | 0.117 |
| Beverage workers | 0.433 | 0.177 | 0.767 | 0.279 |
| Tobacco workers | 0.311 | 0.252 | –0.102 | 0.420 |
| Glass makers etc | 0.121 | 0.096 | 0.124 | 0.123 |
| Packers | 0.207 | 0.074 | 0.389 | 0.076 |
| Engine operators | 0.232 | 0.089 | –0.228 | 0.123 |
| Public safety workers | 0.097 | 0.101 | 0.010 | 0.135 |
| Cooks and stewards | 0.369 | 0.128 | 0.739 | 0.162 |
| Waiters | 0.326 | 0.158 | 1.441 | 0.181 |
| Building caretakers | 0.273 | 0.097 | –0.093 | 0.141 |
| Chimney sweeps | 0.087 | 0.204 | 0.185 | 0.335 |
| Hairdressers | 0.189 | 0.163 | 0.345 | 0.259 |
| Launderers | 0.452 | 0.178 | –0.201 | 0.344 |
| Military personnel | 0.052 | 0.114 | –0.132 | 0.162 |
| Other workers | 0.075 | 0.108 | 0.521 | 0.126 |
| Economically inactive | 0.217 | 0.057 | 0.753 | 0.046 |

**Supplementary Table 2** Standardised incidence ratios (SIR) for lung cancer and larynx cancer, respectively, according to occupational group among 893 264 men from the Norwegian 1970 census, followed 1971–1991. Observed numbers (Obs) and original SIR (denoted ‘SIR’ in the table) were taken from a published study (Andersen *et al*, 1999 [2]), while additional adjustment (adjSIR) was obtained in the present study for tobacco smoking, applied to the lung cancer SIRs; and for smoking and alcohol drinking, applied to the larynx cancer SIRs. Confidence intervals (95% CI) were derived assuming a Poisson distribution of observed cases

|  | Lung cancer | | | |  | Larynx cancer | | | |
| --- | --- | --- | --- | --- | --- | --- | --- | --- | --- |
| Occupational group | Obs | SIR | adjSIR | 95% CI |  | Obs | SIR | adjSIR | 95% CI |
|  |  |  |  |  |  |  |  |  |  |
|  |  |  |  |  |  |  |  |  |  |
| Technical workers, etc | 321 | 0.64 | 1.02 | 0.91, 1.14 |  | 33 | 0.63 | 1.00 | 0.69, 1.41 |
| Laboratory assistants | 28 | 0.93 | 1.07 | 0.71, 1.55 |  | 1 | 0.33 | 0.33 | 0.01, 1.82 |
| Physicians | 32 | 0.49 | 0.85 | 0.58, 1.20 |  | 3 | 0.46 | 0.50 | 0.10, 1.45 |
| Dentists | 20 | 0.73 | 0.99 | 0.60, 1.53 |  | 2 | 0.72 | 0.88 | 0.11, 3.17 |
| Assistant nurses | 10 | 0.76 | 0.84 | 0.40, 1.54 |  | 2 | 1.53 | 1.28 | 0.15, 4.61 |
| Other health workers | 23 | 0.65 | 0.89 | 0.57, 1.34 |  | 3 | 0.86 | 0.69 | 0.14, 2.03 |
| Teachers | 168 | 0.46 | 0.86 | 0.73, 1.00 |  | 15 | 0.40 | 0.65 | 0.36, 1.07 |
| Religious workers etc | 126 | 0.59 | 0.91 | 0.76, 1.08 |  | 13 | 0.62 | 0.64 | 0.34, 1.09 |
| Artistic workers | 41 | 0.83 | 0.88 | 0.63, 1.19 |  | 5 | 0.99 | 0.57 | 0.18, 1.32 |
| Journalists | 21 | 0.76 | 0.89 | 0.55, 1.36 |  | 1 | 0.36 | 0.41 | 0.01, 2.29 |
| Administrators | 713 | 0.81 | 0.95 | 0.88, 1.02 |  | 96 | 1.10 | 1.13 | 0.92, 1.38 |
| Clerical workers | 579 | 0.86 | 1.08 | 0.99, 1.17 |  | 64 | 0.94 | 0.94 | 0.72, 1.20 |
| Sales agents | 605 | 0.95 | 0.87 | 0.81, 0.95 |  | 90 | 1.41 | 1.31 | 1.06, 1.62 |
| Shop workers | 296 | 0.98 | 0.98 | 0.87, 1.10 |  | 27 | 0.88 | 0.86 | 0.57, 1.26 |
| Farmers | 755 | 0.42 | 0.84 | 0.78, 0.90 |  | 59 | 0.34 | 0.75 | 0.57, 0.96 |
| Gardeners | 127 | 0.69 | 1.01 | 0.85, 1.21 |  | 7 | 0.39 | 0.56 | 0.23, 1.15 |
| Fishermen | 436 | 1.16 | 0.72 | 0.65, 0.79 |  | 28 | 0.75 | 0.95 | 0.63, 1.38 |
| Forestry workers | 88 | 0.47 | 0.95 | 0.76, 1.17 |  | 9 | 0.50 | 0.71 | 0.32, 1.34 |
| Miners and quarry workers | 162 | 1.39 | 1.03 | 0.88, 1.20 |  | 9 | 0.77 | 0.85 | 0.39, 1.61 |
| Seamen | 585 | 1.69 | 1.08 | 0.99, 1.17 |  | 70 | 1.93 | 1.25 | 0.98, 1.58 |
| Transport workers | 275 | 0.90 | 0.89 | 0.79, 1.00 |  | 29 | 0.95 | 0.93 | 0.62, 1.34 |
| Drivers | 940 | 1.38 | 1.18 | 1.11, 1.26 |  | 91 | 1.31 | 1.11 | 0.89, 1.36 |
| Postal workers | 107 | 0.79 | 0.82 | 0.67, 0.99 |  | 13 | 0.97 | 1.05 | 0.56, 1.79 |
| Textile workers | 103 | 0.74 | 0.87 | 0.71, 1.06 |  | 14 | 1.03 | 1.27 | 0.70, 2.14 |
| Shoe and leather workers | 36 | 0.89 | 0.92 | 0.64, 1.27 |  | 4 | 1.01 | 0.98 | 0.27, 2.50 |
| Smelting workers | 326 | 1.39 | 1.21 | 1.09, 1.35 |  | 24 | 1.03 | 0.97 | 0.62, 1.45 |
| Mechanics | 1254 | 1.31 | 1.06 | 1.00, 1.12 |  | 114 | 1.16 | 1.12 | 0.92, 1.34 |
| Plumbers | 153 | 1.34 | 1.14 | 0.97, 1.34 |  | 11 | 0.93 | 0.81 | 0.41, 1.45 |
| Welders | 211 | 1.31 | 0.96 | 0.83, 1.09 |  | 15 | 0.90 | 1.01 | 0.56, 1.66 |
| Electrical workers | 429 | 1.06 | 1.14 | 1.03, 1.25 |  | 41 | 0.98 | 1.22 | 0.88, 1.66 |
| Wood workers | 1118 | 0.83 | 0.95 | 0.89, 1.01 |  | 111 | 0.84 | 1.09 | 0.90, 1.31 |
| Painters | 260 | 1.38 | 1.20 | 1.06, 1.36 |  | 19 | 1.02 | 0.93 | 0.56, 1.45 |
| Other construction workers | 596 | 1.05 | 1.06 | 0.98, 1.15 |  | 52 | 0.95 | 1.17 | 0.87, 1.54 |
| Bricklayers | 162 | 1.26 | 1.04 | 0.88, 1.21 |  | 14 | 1.10 | 0.91 | 0.50, 1.52 |
| Printers | 122 | 1.18 | 0.97 | 0.81, 1.16 |  | 9 | 0.86 | 0.69 | 0.32, 1.31 |
| Chemical process workers | 367 | 1.26 | 1.18 | 1.06, 1.30 |  | 41 | 1.43 | 1.20 | 0.86, 1.63 |
| Food workers | 342 | 1.20 | 1.02 | 0.91, 1.13 |  | 43 | 1.52 | 1.54 | 1.11, 2.07 |
| Beverage workers | 30 | 1.92 | 1.22 | 0.82, 1.74 |  | 2 | 1.30 | 0.71 | 0.09, 2.55 |
| Tobacco workers | 8 | 2.12 | 1.52 | 0.66, 3.00 |  | 1 | 2.71 | 2.80 | 0.07, 15.6 |
| Glass makers etc | 286 | 1.30 | 1.13 | 1.00, 1.27 |  | 28 | 1.27 | 1.11 | 0.74, 1.61 |
| Packers | 701 | 1.36 | 1.08 | 1.00, 1.17 |  | 77 | 1.52 | 1.09 | 0.86, 1.36 |
| Engine operators | 369 | 1.29 | 1.00 | 0.90, 1.11 |  | 26 | 0.88 | 1.00 | 0.65, 1.47 |
| Public safety workers | 200 | 1.02 | 0.91 | 0.79, 1.04 |  | 25 | 1.29 | 1.22 | 0.79, 1.81 |
| Cooks and stewards | 92 | 1.48 | 1.00 | 0.81, 1.23 |  | 16 | 2.46 | 1.37 | 0.78, 2.22 |
| Waiters | 45 | 1.66 | 1.18 | 0.86, 1.57 |  | 5 | 1.81 | 0.60 | 0.19, 1.40 |
| Building caretakers | 253 | 1.27 | 0.95 | 0.84, 1.07 |  | 21 | 1.09 | 1.12 | 0.69, 1.71 |
| Chimney sweeps | 14 | 1.09 | 0.98 | 0.54, 1.65 |  | 1 | 0.78 | 0.65 | 0.02, 3.64 |
| Hairdressers | 35 | 1.27 | 1.03 | 0.71, 1.43 |  | 5 | 1.83 | 1.36 | 0.44, 3.18 |
| Launderers | 27 | 1.72 | 1.07 | 0.71, 1.56 |  | 1 | 0.65 | 0.72 | 0.02, 4.00 |
| Military personnel | 130 | 0.99 | 0.92 | 0.77, 1.10 |  | 13 | 0.93 | 0.98 | 0.52, 1.67 |
| Other workers | 194 | 1.34 | 1.22 | 1.05, 1.40 |  | 25 | 1.72 | 1.12 | 0.73, 1.66 |
| Economically inactive | 1549 | 1.41 | 1.11 | 1.05, 1.17 |  | 153 | 1.47 | 0.80 | 0.68, 0.94 |
|  |  |  |  |  |  |  |  |  |  |
